# Improved reverse transcription-polymerase chain reaction assay for the detection of flaviviruses with semi-nested primers for discrimination between dengue virus serotypes and Zika virus

**DOI:** 10.1590/0074-02760170393

**Published:** 2018-02-26

**Authors:** Allan RD Nunes, Brenda Elen B Alves, Hannaly WB Pereira, Yasmin M Nascimento, Ingryd C Morais, José Veríssimo Fernandes, Josélio MG Araújo, Daniel CF Lanza

**Affiliations:** 1 Universidade Federal do Rio Grande do Norte Universidade Federal do Rio Grande do Norte Departamento de Bioquímica Laboratório de Biologia Molecular Aplicada NatalRN Brasil Universidade Federal do Rio Grande do Norte, Departamento de Bioquímica, Laboratório de Biologia Molecular Aplicada, Natal, RN, Brasil; 2 Universidade Federal do Rio Grande do Norte Universidade Federal do Rio Grande do Norte Departamento de Microbiologia e Parasitologia Laboratório de Biologia Molecular de Doenças Infecciosas e Câncer NatalRN Brasil Universidade Federal do Rio Grande do Norte, Departamento de Microbiologia e Parasitologia, Laboratório de Biologia Molecular de Doenças Infecciosas e Câncer, Natal, RN, Brasil; 3 Universidade Federal do Rio Grande do Norte Universidade Federal do Rio Grande do Norte NatalRN Brasil Universidade Federal do Rio Grande do Norte, Programa de Pós-Graduação em Bioquímica, Natal, RN, Brasil; 4 Universidade Federal do Rio Grande do Norte Universidade Federal do Rio Grande do Norte NatalRN Brasil Universidade Federal do Rio Grande do Norte, Programa de Pós-Graduação em Biologia Parasitária, Natal, RN, Brasil; 5 Universidade Federal do Rio Grande do Norte Universidade Federal do Rio Grande do Norte Instituto de Medicina Tropical Laboratório de Virologia NatalRN Brasil Universidade Federal do Rio Grande do Norte, Instituto de Medicina Tropical, Laboratório de Virologia, Natal, RN, Brasil

**Keywords:** arbovirus, Flaviviridae, West Nile virus, yellow fever virus

## Abstract

**BACKGROUND:**

The genus *Flavivirus* includes a variety of medically important viruses, including dengue virus (DENV) and Zika virus (ZIKV), which are most prevalent in Brazil. Because the clinical profile of patients affected by different DENV serotypes or ZIKV may be similar, the development of new methods that facilitate a rapid and accurate diagnosis is crucial.

**OBJECTIVES:**

The current study aimed to develop an improved reverse transcription-polymerase chain reaction (RT-PCR) protocol for universal detection of flaviviruses by using semi-nested primers that discriminate between DENV serotypes and ZIKV.

**METHODS:**

The bioinformatics workflow adopted for primer design included: (1) alignment of 1,442 flavivirus genome sequences, (2) characterisation of 27 conserved regions, (3) generation of a primer set comprising 77 universal primers, and (4) selection of primer pairs with greatest coverage and specificity. Following primer design, the reaction was validated *in vitro*. The same approach was applied to the design of primers specific for DENV and ZIKV, using a species-specific sequence database.

**FINDINGS:**

The new assay amplified an 800-806 nt variable region of the NS5 gene and allowed discrimination of virtually all flavivirus species using reference-sequence comparison. The 800-806 nt fragment was validated as a template for a semi-nested multiplex PCR using five additional primers for the detection of DENV and ZIKV. These primers were designed to generate amplicons of different sizes, allowing differentiation of the four serotypes of DENV, and ZIKV using agarose gel electrophoresis.

**MAIN CONCLUSIONS:**

The bioinformatics pipeline allowed efficient primer design, making it possible to identify the best targets within the coding region of the NS5 protein. The multiplex system proved effective in differentiation of DENV1-4 and ZIKV on a 2% agarose gel. The possibility of discriminating DENV serotypes and ZIKV in the same reaction provided a faster result consuming less sample. In addition, this simplified approach ensured the reduction of the cost per analysis.

The genus *Flavivirus* (family Flaviviridae) is comprised of 53 virus species (Simmonds et al. 2017), including human pathogens such as West Nile virus, Japanese encephalitis virus, tick-borne encephalitis virus, yellow fever virus, dengue virus (DENV) and Zika virus (ZIKV) that infect populations of several countries (Mackenzie et al. 2004, Guzman et al. 2010).

Dengue and Zika infections are major problems faced by the Brazilian public health today. DENV has been considered a significant problem since 1986, when serotype 1 (DENV-1) was identified in the state of Rio de Janeiro (Schatzmayr et al. 1986). The serotypes DENV-2 and DENV-3 were introduced in Brazil via Rio de Janeiro in 1990 and 2000, respectively. In subsequent years, these DENV serotypes spread throughout the country reaching 25 of the 26 Brazilian states by the end of 2006 (Nogueira et al. 2007). Serotype DENV-4 re-emerged in Brazil in the Roraima state of the Amazon region in 2010, 28 years after the first outbreak was reported. Following reports of DENV-4 in Roraima in 1982 and 2010, the virus was identified in 2011 in other states of the northern region of Brazil including Amazonas, Amapá, and Pará. DENV-4 was actually detected serologically in populations from several Brazilian states (Nogueira et al. 2011).

In early 2015, Brazil’s Ministry of Health confirmed autochthonous cases of ZIKV infection in the country from positive samples from the states of Bahia and Rio Grande do Norte (Campos et al. 2015, Zanluca et al. 2015). In these recent outbreaks, the illness manifested dengue-like symptoms, characterised by bloodshot eyes, fever, joint pain, headache, and a typical flat pinkish rash (DuPont-Rouzeyrol et al. 2015, Calvet et al. 2016). These characteristics, associated with the fact that many arboviruses may co-circulate in the same area and co-infect the same patient (DuPont-Rouzeyrol 2015, Villamil-Gómez et al. 2016), pose a challenge for medical diagnosis and patient management.

Currently, the laboratory diagnosis of diseases caused by flaviviruses is carried out using specific serological assays, commonly based on enzyme-linked immunosorbent assays (ELISAs). These tests detect virus-specific IgM and IgG antibodies 5-7 days after onset of infection, which makes it unfeasible for a rapid diagnosis in most cases. In contrast, polymerase chain reaction (PCR) and it derivations can be used during the acute phases of infection and are known to be rapid, specific, and capable of pathogen detection with a great sensitivity. Several reported PCR assays for flaviviruses are only of use as confirmatory tests after the clinical testing is completed and the differential diagnosis made because the PCR primers may only amplify one, or a limited range of closely related species (Fulop et al. 1993, Tanaka 1993, Pierre et al. 1994).

Since the publication of the first tests using universal primer pairs for flaviviruses in 1993 (Fulop et al. 1993, Tanaka 1993), several universal primer sets have been developed for the same purpose (Eldadah et al. 1991, Chow et al. 1993, Pierre et al. 1994, Puri et al. 1994, Figueiredo et al. 1998, Kuno 1998, Scaramozzino et al. 2001, Sánchez-Seco et al. 2005, Chao et al. 2007, Dyer et al. 2007, Maher-Sturgess et al. 2008). The most promising PCR protocols for detecting a wide variety of virus species have been those that use primers designed to target conserved genomic regions. These approaches usually comprise an initial step in which a broad range of primers are used to amplify a potential broad range of targets, followed by a nested step, which generates a species-specific amplicon identified by nucleotide sequencing (Kuno 1998, Maher-Sturgess et al. 2008).

The large number of viral sequences deposited every year, in conjunction with the enhancement of bioinformatics tools, has allowed improvement of the traditional strategies in both efficiency and cost of development. In the current work, we implemented a robust bioinformatics approach to identify conserved regions in flaviviruses, comparing 1,442 full genomes of different species. The conserved regions were mapped and primer sets designed, which were submitted to successive selection steps in order to find the best combination for a universal detection system. The final primer pair selected was used to validate a universal PCR protocol able to detect and discriminate virtually all flaviviruses. This protocol was adopted as the first step in a semi-nested RT-PCR approach for the specific detection of DENV1-4 and ZIKV.

## MATERIALS AND METHODS

*Sequence selection and alignment* - Initially, 1,740 nucleotide sequences from 16 different flavivirus species and serotypes were selected from Genbank (http://www.ncbi.nlm.nih.gov/). The initial set included all sequences containing more than 10,000 nt that had been deposited up to April 4, 2016. In cases where the number of sequences of a single species was greater than 300, the initial set was restricted to sequences deposited from January 1, 2014 or January 1, 2015. Sequences manually excluded that contained regions with inconclusive or degenerate bases, or sequences of virus-attenuated vaccines, clones, and virus chimeras. The predicted amino-acid sequences encoded by the 1,442 selected genomes were generated using Geneious software, version 9.1 (http://www.geneious.com), and aligned using MAFFT algorithm, version 7.222 adopting standard parameters for each case. When necessary, adjustments were made manually.

*Virus samples* - The DENV1-4 and ZIKV samples used for the *in vitro* tests were isolated from patients from the Brazilian states of Rio Grande do Norte (DENV1-4) and Pernambuco (ZIKV), with a confirmed positive-diagnosis in each case. The established kidney epithelial VERO E6 cell-line from an African green monkey was cultured in 12.5 cm^2^ flasks (Corning Incorporated, Corning, New York, USA) in the presence of Leibovitz-15 (L-15) medium (Invitrogen, New York, USA), supplemented with 10% heat inactivated foetal bovine serum (FBS), 1% antibiotic-antimycotic, and 10% tryptose phosphate (Invitrogen, New York, USA). The cell culture was maintained in an incubator at 37ºC in 5% CO_2_. When the cell monolayers reached 90% confluency, 15 μL of the patient serum and 50 μL of 2% FBS L-15 medium were added. The serum-exposed cells were kept in the incubator for 90 min with slight shaking every 30 min. The inoculum was then removed and 3 mL of 2% L-15 medium was added to the flask. The cells were incubated at 37ºC in 5% CO_2_ for seven days to allow for virus replication. For each 1-mL of the cell-culture supernatant, 0.9 mL of bovine serum albumin (BSA) was added to preserve the viral particles. This mixture was centrifuged at 1,500 rpm (~260 ×*g*) for 5 min and the supernatant aliquoted into cryogenic tubes and stored at -70ºC. Confirmation of viral isolation was performed using a reverse transcription PCR assay (RT-PCR) using RNA extracted from the VERO-culture supernatant as the template.

*Cell culture and replication of DENV and ZIKV* - For viral replication, 100 μL of each viral inoculum was added to VERO E6 cell-cultures previously conditioned in 25 cm^3^ bottles. Cells were incubated for 1 h at 37ºC, being homogenised every 15 min. L-15 media with 2% FBS was added to the cells and incubated at 37ºC for seven days. Viral infection was confirmed by RT-PCR. After confirmation of infection, 20% FBS was added and the isolates stored at -70ºC. The titre of ZIKV was determined by plaque assay to be 1.6 × 10^6^ PFU/mL for ZIKV. Owing to the inherent dilution of each reaction, the maximum concentration used per reaction was 6.2 × 10^3^ PFU. The presence of spiked chikungunya virus in the templates was used in the specificity tests and was confirmed by real time PCR, as described by Lanciotti et al. (2007).

*RNA extraction and PCR parameters* - Viral RNA was extracted from the cell culture supernatant using a QIAamp® Viral RNA Kit (QIAGEN®, California, USA) according to the manufacturer’s protocol. The nested RT-PCR was performed using the Access RT-PCR A1702 Kit (Promega) with slight modification of the manufacturer’s protocol. A pre-treatment step was added to improve primer annealing. Thus, prior to the initiation of reverse transcription and the first amplification, 1 μL of RNA template and different concentrations of flavivirus primers CRNS5_3F1 and CRNS5_7R6 (10-100 pmol each) were mixed and heated at 70ºC for 5 min, followed by an ice-bath incubation for another 5 min. Thereafter, 10 μL of 2× master mix (*Tfl* DNA Polymerase, dNTPs, magnesium sulphate and reaction buffer) and 0.4 μL (2 units) of AMV Reverse Transcriptase were added with the final volume adjusted to 20 μL with deionised water. The PCR reaction cycling was performed on a Bioer Life Touch thermal cycler with an initial incubation at 45ºC for 1 h, followed by 40 cycles of incubation at 95ºC for 2 min, for denaturation at 95ºC for 45 s, annealing at 45ºC for 45 s, and elongation at 63ºC for 1 min. A final extension was performed at 63ºC for 5 min.

For the semi-nested component of the protocol, the primer concentrations were standardised using a primer-concentration gradient for each primer combination. As template, 1 μL of cDNA from the first PCR amplification was mixed with 10 μL of 2× master mix and different concentrations of primer (10-100 pmol each) with the final volume of the reaction mix being adjusted to 20 μL with deionised water. The PCR thermal cycling parameters were incubation at 95ºC for 2 min, followed by 40 sequential cycles of denaturation at 95ºC for 45 s, annealing at 53ºC for 45 s, and elongation at 63ºC for 1 min. Again, a final extension was performed at 63ºC for 5 min. After determining the optimal primer concentrations using an annealing temperature gradient (45-60ºC), the PCR amplification was carried out using the ZIKV and DENV (1-4) samples as the template with multiplexed or isolated primer-pairs.

PCR reactions intended to detect more than one virus type/strain simultaneously, and serial-dilution tests were performed using the parameters described for the first and nested components of the protocol. The nested step of the amplification protocol was performed using the hexaplex-containing forward primers DENV1F6.2, DENV2F10, DENV3F6.1, DENV4F3, and ZIKVF8 in combination with reverse primer CRNS5_7R6.

## RESULTS

*Development of a PCR protocol for the detection of flaviviruses* - A consensus sequence containing 13.7% of identical sites and 60.8% identity was generated from the alignment of the 1,442 selected amino acid sequences referenced in Table I. Regions with at least six conserved amino acids (99% identity per site) and global identity equal to or higher than 95% were considered conserved regions (CRs). Of the 27 CRs selected, 16 were located in the NS5 protein, seven in the NS3 protein, and two in each of the Envelope and NS1 proteins. The flavivirus CRs are characterised in

Supplementary data(Table I).

**TABLE I:** Flaviviruses sequence sets used in this study

Keywords	Search Period	Number of sequences obtained	Number of selected sequences
Dengue virus 1	01.2014 - 04.2016	294	272
Dengue virus 2	01.2014 - 04.2016	244	215
Dengue virus 3	01.2014 - 04.2016	173	163
Dengue virus 4	Until 04.2016	219	170
Ilheus virus	Until 04.2016	3	2
Japanese encephalitis virus	Until 04.2016	239	203
Langat virus	Until 04.2016	4	3
Louping ill virus	Until 04.2016	6	5
Murray Valley encephalitis virus	Until 04.2016	20	12
Rocio virus	Until 04.2016	1	1
Spondweni virus	Until 04.2016	2	1
St. Louis encephalitis virus	Until 04.2016	36	30
Tick-borne encephalitis virus	Until 04.2016	160	137
West Nile virus	01.2015 - 04.2016	161	126
Yellow fever virus	Until 04.2016	95	40
Zika virus	Until 04.2016	83	62

Total	-	1740	1442

A primer set containing 77 primers that potentially aligned in each of the conserved regions was designed [

(Table II)]. These primes were expected to function as universal primers with the variable sites within each sequence being replaced with degenerate nucleotide bases. Eleven primers with the lowest degeneracy (≤ 1000 possible combinations), compatible melting temperatures (average Tm ranging from 50-60ºC), and size ranging from 20 to 25 nt were selected from the primer set [

(Table II) in bold]. Subsequently, the degeneracy of each of the 11 primers was reduced by the insertion of inosines to substitute the most variable sites, but always preserving at least two nucleotides on the 5’ and 3’ ends, and at least 18 specific nucleotides in each sequence.

**TABLE II:** Primers designed and validated in this study

Primer name	Sequence (5’→3’)	Lenght	Tm (°C)	Tm mean (°C)	Degeneracy	Self-dimer	Specificity (nº of seqs in which the primer anneals / nº of seqs tested )	Amplicon size (bp)
CRNS5_3F1	AAYTCNAMNSAYGARATGTA	20	46.8 - 58.1	52,45	32	Not tested	Flaviviruses (1442/1442)	800-806
CRNS5_7R6	CCNARCCACATRWACCADAT	20	52.1 - 60.0	56,05	24	Not tested		
DENV1F6.2	ACTCAGCAAAAGARGCAGTGG	21	59.0 - 60.8	59,9	2	Low propensity	DENV1 (272/272)	181
DENV2F10	TTYRCAAGAAARGTGAGAAG	20	48.8 - 56.0	52,4	8	None	DENV2 (215/215)	245
DENV3F6.1	GAACCAGAAACACCCAAYATGGA	23	58.7 - 61.3	60	2	None	DENV3 (163/163) and DENV1 (2/272)	638
DENV4F3	CACCARGAAGGRAAATGTGAATC	23	56.3 - 59.6	57,9	4	None	DENV4 (170/170)	116
ZIKVF8	GCAATATTTGAAGAGGAAAAAGA	23	53.6	53.6	1	None	ZIKV (62/62)	209

*In vitro validation of the first step of the PCR protocol* - Primers CRNS5_3F1 and CRNS5_7R6 (Table II,

(Table II)] were selected for the *in vitro* validation. These primers annealed to conserved-regions 3 and 7, respectively, amplifying a variable region of 800-806 nt within the NS5 coding sequence. This region enabled the phylogenetic classification of virtually all the flavivirus groups [

(Figure)]. The specificity of the primers was validated by *in silico* PCR analysis using the Geneious software. All sequences used in the testing of specificity are described in Table III. The *in silico* specificity test was performed at different levels of stringency. It was observed that nonspecific annealing was highly unlikely, occurring only when considering an allowance of five or more mismatches.

**TABLE III:** Sequences used in the specificity test

Virus taxa	Number of allowed mismatches					

	0	1	2	3	4	5
Bunyaviridae						
Nairovirus						
Crimean-Congo hemorrhagic fever virus Segment S - NC_005302.1 Segment M - NC_005300.2 Segment L - NC_005301.3	No	No	No	No	No	Yes
Orthobunyavirus						
Oropouche virus Segment S - NC_005777.1 Segment M - NC_005775.1 Segment L - NC_005776.1	No	No	No	No	No	Yes
Phlebovirus						
Candiru virus Segment S - NC_015375.1 Segment M - NC_015373.1 Segment L - NC_015374.1	No	No	No	No	Yes	Yes
Rift Valley fever virus Segment S - NC_014395.1 Segment M - NC_014396.1 Segment L - NC_014397.1	No	No	No	No	No	Yes
Flaviviridae						
Hepacivirus						
Hepatitis C virus genotype 1 NC_004102.1	No	No	No	No	No	No
Hepatitis C virus genotype 2 NC_009823.1	No	No	No	No	No	Yes
Hepatitis C virus genotype 3 NC_009824.1	No	No	No	No	No	Yes
Hepatitis C virus genotype 4 NC_009825.1	No	No	No	No	No	Yes
Hepatitis C virus genotype 5 NC_009826.1	No	No	No	No	No	Yes
Hepatitis C virus genotype 6 NC_009827.1	No	No	No	No	No	Yes
Hepatitis C virus genotype 7 NC_030791.1	No	No	No	No	No	Yes
Pestivirus						
Bovine viral diarrhea virus genotype 1 NC_001461.1	No	No	No	No	No	No
Bovine viral diarrhea virus genotype 2 NC_002032.1	No	No	No	No	No	Yes
Orthomyxoviridae						
Influenzavirus A						
Influenza A virus (H1N1) Segment 1 - NC_026438.1 Segment 2 - NC_026435.1 Segment 3 - NC_026437.1 Segment 4 - NC_026433.1 Segment 5 - NC_026436.1 Segment 6 - NC_026434.1 Segment 7 - NC_026431.1 Segment 8 - NC_026432.1	No	No	No	No	No	Yes
Rabdoviridae						
Vesiculovirus						
Chandipura virus NC_020805.1	No	No	No	No	No	Yes
Vesicular stomatitis Indiana virus NC_001560.1	No	No	No	No	No	Yes
Vesicular stomatitis New Jersey virus NC_024473.1	No	No	No	No	No	Yes
Vesicular stomatitis Alagoas virus NC_025353.1	No	No	No	No	No	Yes
Reoviridae						
Orbivirus						
Changuinola virus Segment 1 - NC_022639.1 Segment 2 - NC_022633.1 Segment 3 - NC_022634.1 Segment 4 - NC_022640.1 Segment 5 - NC_022635.1 Segment 6 - NC_022641.1 Segment 7 - NC_022636.1 Segment 8 - NC_022637.1 Segment 9 - NC_022642.1 Segment 10 - NC_022638.1	No	No	No	No	No	Yes
Togavidae						
Alphavirus						
Barmah Forest virus NC_001786.1	No	No	No	No	No	Yes
Chikungunya virus NC_004162.2	No	No	No	No	No	Yes
Mayaro virus NC_003417.1	No	No	No	No	No	Yes
Ross River virus NC_001544	No	No	No	No	No	Yes
Sindbis virus NC_001547.1	No	No	No	No	Yes	Yes

**Fig. 1:**
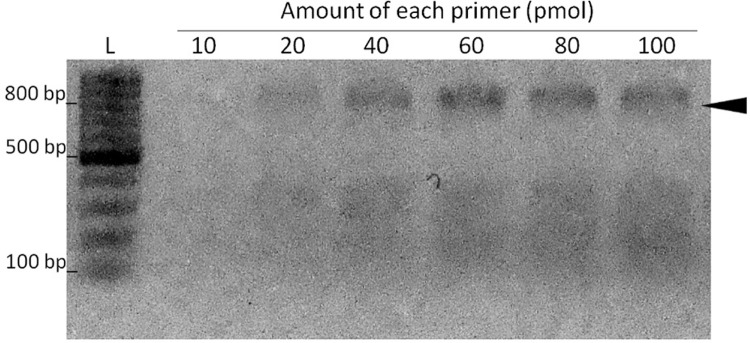
: effect of primer concentration on the reverse transcription-polymerase chain reaction (RT-PCR) for flavivirus detection. Six reactions were carried out using a temperature of 45ºC for annealing, and 63ºC for extension with Zika virus (ZIKV) cDNA as the template in order to determine the best primer concentration for the use of degenerate primers CRNS5_3F1 and CRNS5_7NR6. The amount of each primer ranged from 10-100 pmol. The amount of each primer used per reaction are presented above each lane (pmol). The black arrow indicates the expected size for the 800-bp amplicon. L = 100-bp size marker.

The standardisation of the PCR reaction using CRNS5_3F1 and CRNS5_7R6 primers was started with gradient tests in order to determine the best annealing and elongation temperatures (data not show). Once the annealing temperature of 45ºC, and the elongation temperatures of 63ºC were determined as optimal, a primer gradient test revealed that 60 pmol of each primer was the ideal amount for the assay, as shown in Fig. 1.

*Development of a semi nested-PCR for the detection of DENV1-4 and ZIKV* - After standardisation of the first step of the PCR reaction, the next challenge was to determine the regions that allowed for sensitive and specific amplification using a multiplex semi-nested PCR. It was anticipated that this second step would generating amplicons of different sizes, allowing the discrimination between the DENV strains 1-4, as well as ZIKV, using only gel electrophoresis to analyse the PCR results.

**Fig. 2:**
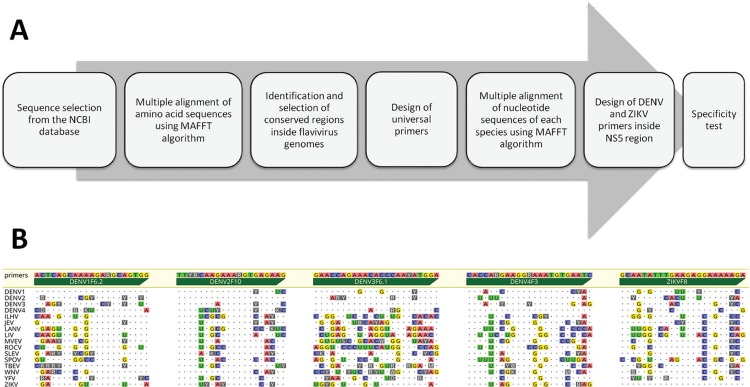
: pipeline for primer design and results from the specificity test. (A) Schematic of the pipeline used for the design and validation of primers. (B) Sequences of the forward primers targeting the flavivirus NS5 coding region to discriminate dengue virus (DENV)1-4 serotypes and Zika virus (ZIKV) in the semi-nested polymerase chain reaction. Primer sequences are presented in the 5’-3’ orientation above the green arrows. Dotted lines correspond to the consensus sequence obtained from the alignment of the sequence sets for each species referenced in Table I. Identities are indicated by dots and mismatches by letters (nucleotide bases or degenerate bases).

To identify the best target sites for primer design, new CRs were identified from alignment of the 882 amino acid sequences of DENV and ZIKV. Subsequently, 14 alignments were performed, one for each sequence set shown in Table I. Conserved regions that existed among the species or serotypes were discarded. Specific CRs located within the NS5 sequence of DENV1-4 and ZIKV genomes were selected as targets for which a new set of 81 candidate primers was designed, based on the following criteria: (1) primer length of 20-25 nucleotides; (2) maximum degeneration of eight combinations; (3) average Tm from 52-60ºC; (4) absence of degeneration in the three nucleotides at 3’ end; (5) the first two nucleotides of the 3’ end should not be identical (coincidental sites) between the ZIKV and DENV sequences; and (6) secondary structure with ΔG > -5 Kcal/mol. The prediction of the secondary structures was performed with the Geneious software, using the DNA model in the toolkit plugin Vienna. The pipeline used for primer design is summarised in Fig. 2A.

Following primer design, the specificity of each primer was analysed by means of *in silico* PCR analysis using the complete flavivirus sequence set as a template. This test revealed that most primers would fail to anneal on a significant number of unspecific targets [

(Table III)], even when adopting a low level of stringency (i.e. allowing for five mismatches). Among the 81 candidate primers, the five forward primers presented in Table II were selected for *in vitro* validation. The result of the *in silico* specificity test for the five selected primers against the sequence set described in Table I is summarised in Fig. 2B.

**Fig. 3:**
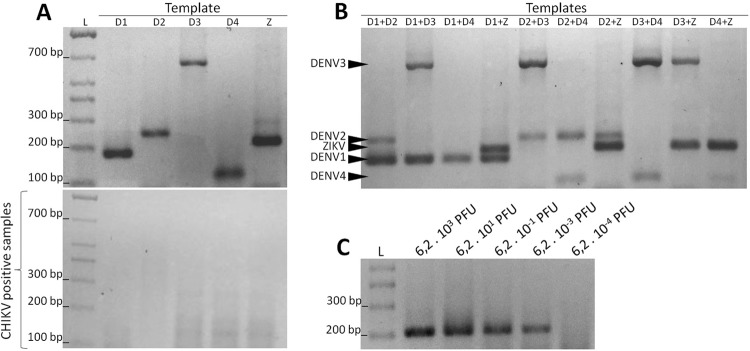
: evaluation of the semi-nested polymerase chain reaction (PCR) for identification of dengue virus (DENV)1-4 serotypes and Zika virus (ZIKV). The efficiency of the semi-nested reaction using the primers DENV1F6.2, DENV2F10, DENV3F6.1, DENV4F3, ZIKVF8, and CRNS5_7NR6 was evaluated under different conditions. (A) Size resolution of each amplicon in a 2% agarose gel. The letters above each lane indicate the templates containing RNA of DENV serotypes (D1, D2, D3, and D4) or ZIKV (Z). The results of semi-nested reactions containing each primer individually and a template positive for Chikungunya virus are presented (below). (B) Tests using a mixture of reverse-transcribed RNAs as the template from two types of viruses. All possible combinations among the five viruses have been evaluated and are shown above each their respective gel lanes. The size of each amplicon in the 2% agarose gel are indicated (black arrows). (C) Dilution test to verify the sensitivity of the semi-nested reaction. L = 100 bp size marker.

*In vitro validation of the semi-nested PCR protocol* - The *in vitro* validation using specific templates confirmed that the multiplex containing the forward primers DENV1F6.2, DENV2F10, DENV3F6.1, DENV4F3, and ZIKVF8, and the reverse primer CRNS5_7R6 produced specific amplicons that were separated by 2% agarose gel electrophoresis (Fig. 3A top). Separate reactions with each of the forward primers using Chikungunya virus template as a positive control confirmed the specificity of the reactions (Fig. 3A down).

The semi-nested PCR was also tested with heterologous templates containing two different serotypes or species of viruses mixed together. The hexaplex reactions efficiently detected all double-combinations tested of the five viruses. As demonstrated in Fig. 3B, any of the DENV1-4 and ZIKV amplicons in the same electrophoresis lane were easily separated in a 2% agarose gel. The test was also able to detect three or more viruses mixed and used as a template, but under these conditions small RNA concentrations could influence the results (data not show).

**Fig. 4:**
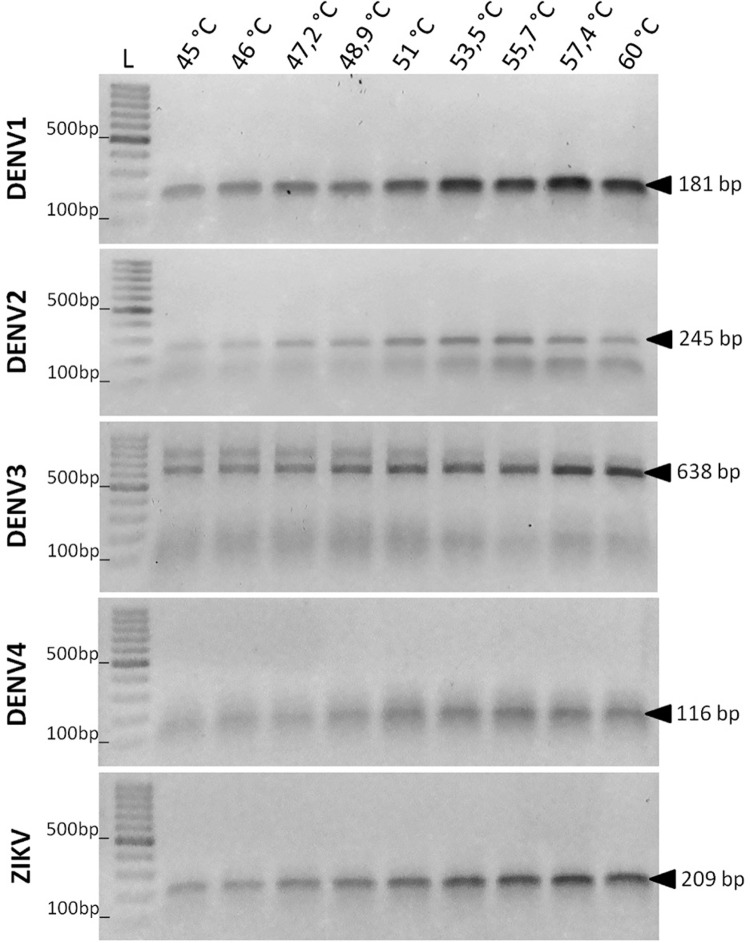
: polymerase chain reaction (PCR) efficiency at different annealing temperatures. The efficiency of the semi-nested PCR reaction containing the six primers was evaluated at different annealing temperatures (45-60ºC) for each of the five viruses (specified on the left). The expected amplicon sizes are indicated (black arrows). L = 100 bp size marker.

The sensitivity of the PCR was also assessed using ZIKV as a template. The serial dilution of the ZIKV template revealed that the nested multiplex reaction was able to detect 6.2 × 10^3^ plaque forming units (PFU), (Fig. 3C). In addition, as shown in Fig. 4, the hexaplex reactions efficiently discriminated all serotypes and species using different annealing temperatures. All PCR-generated amplicons were sequenced by Sanger methodology in order to confirm the specificity of each reaction (data not show).

## DISCUSSION

The biggest challenge in the development of universal primers was to define conserved regions suitable for the primer targeting. The analysis of 1,442 flaviviruses sequences allowed for an accurate characterisation of 27 conserved regions. Application of the pipeline with successive steps involving specific selection criteria identified from the regions CRNS5_3 and CRNS5_7 as the most promising for the designing of universal primers. In agreement, Maher-Sturgess et al. (2008), coincidentally identified these regions from the alignment of 257 full-length flavivirus genomes. These regions were set as targets by these investigators for the design of primers that amplify equivalent fragments of NS5, allowing the phylogenetic reconstruction of each flavivirus subgroup. In our study, the alignment of a greater number of sequences assured the improvement of the universal primers, mainly by the elimination of potential mismatches, increasing the possibility of amplification of templates with a greater sequence diversity.

A main advantage of the *in silico* approach was the considerable reduction in the time spent to validate the *in vitro* protocol. In fact, only the primer concentrations, and the annealing and elongation temperatures required optimising, and without the need for additives or different mixtures in order to achieve a high specificity. As expected, the concentration of primers was critical to the efficiency of the reaction; for the first step, higher primer concentrations provided the best results. This may have been attributed to the degeneracy of the primers in association with a single type of virus DNA used as a template. In the multiplex reaction, this effect was less pronounced because of the lower degeneration of the primers. In both the first PCR reaction and the nested reaction, the *in vitro* results corroborated the efficiency of the *in silico* analyses to predict specificity and the ideal annealing conditions. The nested multiplex reaction produced specific amplicons, even when using templates that contained more than one type of virus, or having different annealing temperatures.

Another point that could be better explored by the *in silico* approach was the analyses regarding specificity. In tropical countries, the circulation of new types of arboviruses is relatively frequent, as well as cases of co-infection. The success of diagnostic PCR assays depends in part on the primers being specific to a particular group of viruses. For most laboratories, it is impracticable to test all sample-possibilities *in vitro* because the storage of these samples is not feasible. The *in silico* approach made it possible to expand the specificity analyses in both interspecific and intraspecific ways. The specificity tests consisted of species from six viral families used for the validation of the primer pair CRNS5_3F1/CRNS5_7R6, and 1,442 viral genomes for the validation of the DENV and ZIKV primers. Thus, it was possible to evaluate the specificity of primers for sequences that had never been included in previous work.

A final point to be discussed is the technical feasibility of the proposed method. To be effective in a clinical setting, the test should be able to detect the pathogen within seven days after the onset of the first symptoms. The method proposed here allowed the investigation of five viruses using a single reaction to discriminate each species by simple gel electrophoresis. In addition, the test was shown to be effective for identifying two different types of virus in the same sample. This is crucial because co-infections are possible, but are rare with more than two types of virus. Another important point is that in the amount of template required for the PCR protocol was small. This is important in the real-world applications because clinical specimens are often limited, for example, volume samples of CSF, or samples from neonates.

*In conclusion* - The *in silico* approach presented here made it possible to accurately determine targets among 1,442 sequences, and to reduce the time required for the development of an effective set of primers. The best target identified was the region encoding the NS5 protein. The efficiency of the primers was confirmed *in vitro*, and the hexaplex system proved to be efficient in detecting and identifying DENV strains 1-4, and ZIKV by analysing the PCR reaction products on a 2% agarose gel. The pipeline presented in this work should be considered as an option to reduce the cost and time for the development of primers to identify other pathogenic species. These features are of value for public health programs, especially in developing countries where DNA sequencing or real time PCR are not feasible.
