# Supplementary material for: Improved reverse transcription-polymerase chain reaction assay for the detection of flaviviruses with semi-nested primers for discrimination between dengue virus serotypes and Zika virus
Source: Mem Inst Oswaldo Cruz. 2018 Feb 26;113(5):e170393. doi: 10.1590/0074-02760170393 (PMC5853760; doi:10.1590/0074-02760170393)
Supplement: Supplementary file 1 [file 0074-0276-mioc-113-5-e170393-suppl01.pdf]

TABLE I
Conserved regions in flavivirus genomes

Protein	CR	Conserved region (amino acid)	Identical sites (%)	Pairwise % Identity	Conserved region (nucleotide 5'-3')
Env					
	CRE_1	DRGWG(N/K)(G/H)C(G/E)(L/F)(F/S)GK(G/R)	57,1%	98,60%	GAYMGNGGNTGGGGNAABSRNTGYGRNYTNTYYGG-NAARRGN
	CRE_2	AWDF(G/S/R)S	83,3%	99,40%	GCNTGGGAYTTYVGNTCN
NS1					
	CRNS1_1	WC(C/S)(R/K)(S/N/E/T/A)C	50,0%	95,50%	TGGTGYTSYMRNDVNTGY
	CRNS1_2	CWY(G/A/R/P/S)(M/V)E(I/V)RP	66,7%	97,40%	TGYTGGTAYBSNRTGGARRTHMGNCCN
NS3					
	CRNS3_1	G(T/S)(S/F)GSP(I/T)	57,1%	99,90%	GGNWSNTYNGGNWSNCCNAYH
	CRNS3_2	(G/S)(L/C)(Y/L/C)GNG	50,0%	99,90%	RGNYKNTDBGGNAAYGGN
	CRNS3_3	L(A/V)P(T/S)R(V/A)(V/G)	42,9%	99,90%	YTNGYNCCNASNMGNGYNGKNBYN
	CRNS3_4	(S/G/R)(I/L/T/V)AARG	66,7%	96,50%	RGBDYNGCNGCNMGNGGN
	CRNS3_5	MTAT(P/S)(P/R)G	71,4%	99,96%	ATGACNGCNACNYCNCSNGGN
	CRNS3_6	(T/A)D(I/M)(S/A)(E/V)(M/V)GA(N/Y)	33,3%	99,30%	RCNGAYATNKCNGWRRTGGGNGCNWAY
	CRNS3_7	AS(A/T/N)(Q/P)RRGR(I/V)(G/S)R	63,6%	95,40%	WSNGCNRMNCMRMGNMGNGGNMGNRTNRGNMG
NS5					
	CRNS5_1	DLGCG(R/C)G(G/S)W(S/C/N)Y(Y/C)	66,7%	98,20%	GAYYTNGGNTGYGGNHGNGGNRGNTGGWVNTAYTRY
	CRNS5_2	(C/Y/G)D(I/V)GES	66,7%	99,90%	KRYGAYRTHGGNGARWSN
	CRNS5_3	P(L/F)(S/C)(R/K)NS(T/N)(H/D)EMY	54,5%	96,00%	CCNYTNTSNMRNAAYTCNAMNSAYGARATGTAY
	CRNS5_4	AM(T/N)DT(T/K)	66,7%	99,95%	GCNATGAMNGAYACNAMN
	CRNS5_5	GQQRVFK(E/D)KVDT	91,7%	99,60%	GGNCARCARMGNGTNTTYAARGANAARGTNGAYACN
	CRNS5_6	(C/G/R)(V/A/I)(Y/H)(N/I/H)(M/T)MG(K/M)REKK	50,0%	96,70%	BGYRYNYAYMWYAYRATGGGNAWRMGNGARAARAAR
	CRNS5_7	EFG(K/R/V)AKGSR(A/V)IW(Y/F)MWLG(A/S) R(F/Y)L(E/D)F(E/K)ALG(F/S)(L/M)N(E/L)D(H/R)W	67,6%	95,20%	GARTTYGGNRDRGCNAARGGNAGYMGNGYNATHTGGT-WYATGTGGYTNGGVRSNMGNTWYYTNGADTTYRARGC-NYTNGGNTYYHTNAAYDWRGAYCRYTGG
	CRNS5_8	(Y/F)ADDTAGWDT	90,0%	99,90%	TWYGCNGAYGAYACNGCHGGNTGGGAYACN
	CRNS5_9	(D/E/A/N)Q(R/I)(G/R)(S/T/I)(G/E/V)(Q/R)V(V/G)TY	36,4%	95,30%	RMHCARMKDSGNWBNGDNCRRGTNGKNACNTAY
	CRNS5_10	L(N/G)T(F/I/Y/L)TN	66,7%	96,00%	YTNRRYACNHWHACNAAY
	CRNS5_11	(S/T/N)GDD(C/S)(V/L/E/A)(V/G)(K/R/E)P	44,4%	97,30%	AVYGGNGAYGAYWGYSHNGKNVRVCCN
	CRNS5_12	(V/G/F)PF(C/G/W)(S/C)(H/N/R/P)(H/R/S)F	37,5%	95,30%	KKNCCNTTYKGBTSNMVYMRYTTY
	CRNS5_13	(Y/N/D)F(H/Q/D)(R/K)R(D/G/Y)(L/Q/M)(R/C)	25,0%	98,50%	DAYTTYSAHMRNMGNKRYHWNHGN
	CRNS5_14	R(T/P)(T/S)WS(I/V/K)H	57,1%	96,10%	MGNMCNWCNTGGWSNRWHCAY
	CRNS5_15	W(M/I)T(T/A/N)(E/G/Q)(D/N/E)(M/R)L	37,5%	99,70%	UGGAUSACNRMNSRRRABAKGYUN
	CRNS5_16	(V/A)WN(R/K)(V/L)W(I/V)	42,9%	99,80%	GYNTGGAAYMRNKTNTGGRTN

CC BY

TABLE II

Universal primes designed in this study

Protein	Name	Primer sequence (5'-3')	Primer size (nt)	Tm (°C) (average)	Degeneracy
Env					
	CRE_1F	TGYGRNYTNTYYGGNAARRG	20	47.6 - 69.6 (58,6)	8192
	CRE_2F	GCNTGGGAYTTYVGNTCN	18	49.1 - 62.5 (55,8)	768
	CRE_2R	NGANCBRAARTCCCANGC	18	49.3 - 63.1 (56,2)	768
NS1					
	CRNS1_1F	TGGTGYTSYMRNDVNTGY	18	46.6 - 68.4 (57,5)	9216
	CRNS1_1R	RCANBHNYKRSARCACCA	18	46.0 - 68.1 (57,0)	9216
	CRNS1_2F1	TGGTAYBSNRTGGARRTHMGNCC	23	54.4 - 72.0 (63,2)	9216
	CRNS1_2R1	GGNCKDAYYTCCAYNSVRTACCA	23	53.9 - 72.0 (62,9)	9216
	CRNS1_2R2	CKDAYYTCCAYNSVRTACCA	20	47.6 - 65.6 (56,6)	2304
	**CRNS1_2R3**	**AYYTCCAYNSVRTACCARCA**	20	49.9 - 65.2 (57,5)	768
NS3					
	CRNS3_1F	GGNWSNTYNGGNWSNCCNAYH	21	53.2 - 72.1 (62,6)	786432
	CRNS3_1R1	TNGGNSWNCCNRANSWNCC	19	53.0 - 69.1 (61,0)	131072
	CRNS3_2F	RGNYKNTDBGGNAAYGGN	18	43.8 - 70.5 (57,1)	36864
	CRNS3_3F	YTNGYNCCNASNMGNGYNGKNBYN	24	52.4 - 81.3 (62,1)	25165824
	CRNS3_3R1	NRVNMCNRCNCKNSTNGG	18	46.5 - 73.4 (59,9)	393216
	CRNS3_4F	RGBDYNGCNGCNMGNGGN	18	51.7 - 77.7 (64,7)	73728
	CRNS3_5F1	ATGACNGCNACNYCNCSNGG	20	58.2 - 71.3 (64,7)	4096
	CRNS3_5R1	CCNSGNGRNGTNGCNGTCAT	20	57.1 - 72.6 (64,8)	4096
	CRNS3_6F1	GAYATNKCNGWRRTGGGNGC	20	50.5 - 68.5 (62,1)	2048
	CRNS3_6R1	TWNGCNCCCAYYWCNGMNATRTC	23	54.2 - 71.1 (62,6)	16384
	CRNS3_6R2	GCNCCCAYYWCNGMNATRTC	20	51.7 - 68.3 (60,0)	2048
	CRNS3_7F1	NGCNRMNCMRMGNMGNGG	18	50.3 - 77.3 (61,1)	65536
	CRNS3_7R1	CKNCCNCKNCKYKGNKYNGC	20	50.7 - 80.9 (65,8)	131072
NS5					
	CRNS5_1F1	TNGGNTGYGGNHGNGGNRGNTGG	23	59.0 - 80.2 (69,6)	49152
	CRNS5_1F2	TGYGGNHGNGGNRGNTGG	18	53.0 - 74.1 (63,5)	3072
	CRNS5_1F3	GAYYTNGGNTGYGGNHGNGG	20	52.8 - 73.1 (62,9)	6144
	CRNS5_1R1	CCNCDNCCRCANCCNARRTC	20	55.6 - 72.3 (63,9)	6144
	CRNS5_1R2	CCANCYNCCNCDNCCRCANCC	21	59.3 - 76.6 (67,9)	12288
	CRNS5_1R3	CCANCYNCCNCDNCCRCA	18	54.3 - 72.4 (63,3)	3072
	CRNS5_2F	KRYGAYRTHGGNGARWSN	18	42.8 - 65.7 (54,2)	12288
	CRNS5_3F1	**AAYTCNAMNSAYGARATGTA**	20	46.8 - 58.1 (52,4)	512
	CRNS5_3R1	TNGARTTNYKNSANARNGG	19	42.3 - 65.1 (53,7)	32768
	**CRNS5_3R2**	**TACATYTCRTSNKTNGARTT**	20	45.0 - 58.3 (51,6)	512
	CRNS5_4F	GCNATGAMNGAYACNAMN	18	45.7 - 60.7 (53,2)	2048
	CRNS5_4R	NKTNGTRTCNKTCATNGC	18	42.8 - 61.2 (52,0)	2048
	CRNS5_5F1	GTNTTYAARGANAARGTNGAYAC	23	47.1 - 61.3 (54,2)	1024
	**CRNS5_5F2**	**GTNTTYAARGANAARGTNGA**	20	43.6 - 57.2 (50,4)	512
	CRNS5_5R1	TCYTTRAANACNCKYTGYTGNCC	23	54.2 - 69.6 (61,9)	2048
	CRNS5_5R2	TTRAANACNCKYTGYTGNCC	20	50.3 - 66.0 (58,1)	1024
	CRNS5_5R3	TTNTCYTTRAANACNCKYTGYTG	23	50.3 - 65.3 (57,8)	2048
	CRNS5_5R4	**TCYTTRAANACNCKYTGYTG**	20	48.1 - 63.0 (55,5)	512
	**CRNS5_6F1**	**ATGGGNAWRMGNGARAARAA**	20	49.0 - 62.8 (55,9)	512
	CRNS5_6F2	AYRATGGGNAWRMGNGARAA	20	47.8 - 65.2 (56,5)	1024
	CRNS5_6R1	TTYTTYTCNCKYWTNCCCAT	20	47.9 - 61.0 (54,5)	512
	CRNS5_7F1	GGNTYYHTNAAYDWRGAY-CRYTGG	24	50.0 - 70.8 (60,6)	36864
	CRNS5_7F2	TNGGNTYYHTNAAYDWRGA	19	39.6 - 62.6 (51,1)	18432

Protein	Name	Primer sequence (5'-3')	Primer size (nt)	Tm (°C) (average)	Degeneracy
	CRNS5_7F3	TTYRARGCNYTNGGNTYYHTNAA	23	48.0 - 69.5 (58,7)	49152
	CRNS5_7F4	TNGADTTYRARGCNYTNGG	19	44.7 - 63.6 (54,1)	3072
	CRNS5_7F5	TGTGGYTNGGVRSNMGNT-WYYTNGA	25	55.7 - 75.5 (65,6)	98304
	**CRNS5_7F6**	**GYNATHTGGTWYATGTG-GYTNGG**	23	51.7 - 65.5 (58,6)	768
	CRNS5_7F7	AGYMGNGYNATHTGGTWYAT-GTGG	24	52.7 - 69.4 (61,0)	1536
	**CRNS5_7F8**	**GNGYNATHTGGTWYATGTGG**	20	47.4 - 61.4 (54,4)	384
	**CRNS5_7F9**	**ATHTGGTWYATGTGGYTNGG**	20	48.6 - 59.5 (54,0)	96
	CRNS5_7R1	CTNCCYTTNGCYHYNCCRAAYTC	23	52.5 - 70.6 (61,5)	6144
	CRNS5_7R2	CTNCCYTTNGCYHYNCCRAA	20	49.2 - 68.5 (58,8)	3072
	CRNS5_7R3	CCYTTNGCYHYNCCRAAYTC	20	48.8 - 67.2 (58,0)	1536
	CRNS5_7R4	ACCADATNRCNCKRCTNC-CYTTNGC	25	56.8 - 74.2 (65,5)	12288
	CRNS5_7R5	CCACATRWACCADATNRCNCKRCT	24	55.1 - 69.1 (62,1)	1536
	**CRNS5_7R6**	**CCNARCCACATRWACCADAT**	20	52.1 - 60.0 (56,0)	96
	CRNS5_7R7	CKNSYBCCNARCCACATRWACCA	23	56.7 - 71.4 (64,0)	3072
	CRNS5_8F1	GAYGAYACNGCHGGNTGGGAYAC	23	58.2 - 71.8 (65,0)	384
	CRNS5_8F2	GCNGAYGAYACNGCHGGNTGGGA	23	62.1 - 75.2 (68,6)	768
	CRNS5_8F3	GAYGAYACNGCHGGNTGGGA	20	56.5 - 69.4 (62,9)	192
	CRNS5_8R1	TCCCANCCDGCNGTRTCRTCNGC	23	62.6 - 75.0 (68,8)	768
	CRNS5_8R2	TCCCANCCDGCNGTRTCRTC	20	57.0 - 69.1 (63,0)	192
	CRNS5_9F1	GNWBNGDNCRRGTNGKNACNTA	22	48.6 - 73.4 (61,0)	589824
	CRNS5_10F	YTNRRYACNHWHACNAAY	18	39.7 - 61.0 (50,3)	36864
	CRNS5_11F1	GAYGAYWGYSHNGKNVRVCC	20	48.8 - 72.7 (60,7)	55296
	CRNS5_11R1	GGBYBNMCNDSRCWRTCRTCNCC	23	56.6 - 77.6 (67,1)	221184
	CRNS5_12F1	CCNTTYKGBTSNMVYMRYTT	20	46.1 - 69.1 (57,6)	36864
	CRNS5_12R1	AARYKRBKNSAVCMRAANGG	20	45.4 - 70.8 (58,1)	36864
	CRNS5_13F	DAYTTYSAHMRNMGNKRYHWN-HGN	24	47.9 - 76.2 (62,0)	21233664
	CRNS5_13R1	CDNWDRYMNCKNYKDTSRAA	20	42.1 - 72.4 (57,2)	884736
	CRNS5_14F1	GNMCNWCNTGGWSNRWHCA	19	51.8 - 66.5 (59,1)	49152
	CRNS5_15F	UGGAUSACNRMNSRRRABAK-GYUN	24	53.4 - 72.2 (62,8)	98304
	CRNS5_15R1	CMUVUYYYSNKYNGUSAUCCA	21	48.9 - 68.1 (58,5)	12288
	CRNS5_16F1	GYNTGGAAYMRNKTNTGG	18	41.8 - 63.4 (52,6)	2048
	CRNS5_16R1	NAYCCANAMNYKRTTCCA	18	43.7 - 61.9 (52,8)	2048

TABLE III

Primer set for dengue virus (DENV) (1-4) and Zika virus (ZIKV) for use with CRNS5_7NR6 reverse primer

Virus	Primer name	Sequence (5'→3')	Lenght	Tm (°C)	Tm mean (°C)	Degeneracy	Hairpin	Self-dimer	Specificity (3 nt na 3' conserved; max de 5 mismatches)	Amplicon (pb)
DENV1										
	DENV1F1	CCAACYATAGAR-GAAGGAA	19	48.2 -53.4	50,8	4	41.2	9.5	DENV2 (1/217), DENV4 (170/170)	-
	DENV1F2	AGAAAAYATGG-AGGRATGC	18	45.9 - 51.3	48,6	4	N	N	DENV3 (163/163)	-
	DENV1F3	TTYACAAT-GRCTCACAGGAA	20	52.0 - 57.3	54,6	4	N	N	DENV2 (217/217), DENV4 (158/170)	713
	DENV1F4	ACATGGGCCTAY-CATGGATCA	21	58.6 - 61.0	59,8	2	66.7	25.2	N	536
	DENV1F5	GAAAAYCAATG-GAACTCAG-CAAAAG	25	57.9 - 59.8	58,8	2	N	0.1	DENV3 (5/163)	194
	DENV1F6	GGAACTCAG-CAAAAGARG-CAGTGGA	25	63.7 - 65.2	64,7	2	37.4	N	-	184
	DENV1F6.1	AACTCAG-CAAAAGARG-CAGTGGA	23	60.9 - 62.6	61,7	2	37.4	1.6	-	182
	DENV1F6.2	ACTCAG-CAAAAGARG-CAGTGG	21	59.0 - 60.8	59,9	2	37.4	1.6	-	181
	DENV1F7	GGATAYATACTYA-GAGACATATCA	24	50.2 - 54.8	52,5	4	N	20.3	N	-
	DENV1F8	CAARTCATATG-CACARATGTG-GCAG	25	60.0 - 62.4	61,2	4	45.7	14.0	N	-
	DENV1R1	TTTTATRTTYTC-TATCCTCTG	21	45.9 - 50.0	47,9	4	N	N	-	219
	DENV1R2	TCRTCATARTGC-CATGTTGA	20	52.6 - 57.0	54,8	4	34.0	4.2	-	305
	DENV1R3	GATGGYT-TRACCTCATATGA	20	49.8 - 54.6	52,2	4	40.2	11.3	N	-
DENV2										
	DENV2F1	ATAGGGGART-CRTCACCAA	19	52.6 - 57.3	54,9	4	53.3	None	DENV1 (72/272), DENV3 (163/163), JEV (201/203), MVEV (1/12), SLEV (28/31), WNV (125/126)	-

↳

Virus	Primer name	Sequence (5'→3')	Lenght	Tm (°C)	Tm mean (°C)	Degeneracy	Hairpin	Self-dimer	Specificity (3 nt na 3' conserved; max de 5 mismatches)	Amplicon (pb)
	DENV2F2	CYGGGAAY-ATAGTGTCATCAG	21	52.9 - 58.2	55,5	4	41.5	None	DENV1 (264/272), DENV3 (53/163), LANV (3/3), TBEV (14/137)	766
	DENV2F3	AAYATAGTGTCAT-CAGTGAAYATGA	25	53.7 - 58.5	56,1	4	35.8	12.1	DENV1 (272/272), WNV (3/126)	761
	DENV2F4	TGAGYGGAGTG-GAAGGAGAA	20	57.6 - 60.5	59,0	2	None	None	DENV1 (270/272), DENV3 (163/163), DENV4 (168/168)	-
	DENV2F5	GCWCGYGAGGCT-GTTGAA	18	57.6 - 61.3	59,4	4	None	26.3	N	176
	DENV2F6	CTYCATCTT-GARGGAAAGT-GTGA	23	56.7 - 60.0	58,3	4	47.7	9.0	DENV4 (139/170)	119
	DENV2F7	GGAGTG-GAAGGAGA-AGGRCTRCAC	24	61.9 - 67.0	64,4	4	39.5	None	DENV1 (194/272), DENV3 (149/163), DENV4 (145/170)	-
	DENV2F8	TGATYAAYA-GATTYACAATGA	21	45.4 - 53.2	49,3	8	33.7	None	DENV1 (3/272), DENV3 (163/163)	724
	DENV2F9	ACTGGATCAG-CATCWTCYAYGG	22	57.4 - 62.4	59,9	8	47.1	10.7	N	500
	DENV2F10	TTYR-CAAGAAARGT-GAGAAG	20	48.8 - 56.0	52,4	8	56.6	None	-	245
	DENV2R1	GAAAT-CATRTTCACTGAT-GACACTA	25	55.0 - 57.4	56,2	2	33.4	1.4	-	71
	DENV2R2	CTTGAAAT-CATRTTCACTGAT-GAC	24	54.1 - 56.6	55,3	2	39.0	0.8	DENV1 (263/272)	74
	DENV2R3	CATYCTTGAAAT-CATRTTCACTG	23	52.0 - 56.2	54,1	4	39.0	None	DENV1 (264/272), DENV3 (162/163)	72
	DENV2R4	TCTGTCATWGC-CATYTGTG	19	52.6 - 55.2	53,9	4	33.3	None	DENV1 (272/272), DENV3 (156/163), DENV4 (109/170)	398
	DENV2R5	TTCCAYTTRTTCT-CATCAGTGA	22	52.9 - 57.3	55,1	4	35.6	2.8	N	623
	DENV2R6	GTYTCACACTTTC-CYTCAAG	20	52.6 - 55.7	54,1	4	None	None	SLEV (12/31)	710
	DENV2R7	TGTTCTCCTTY-CATRTGGTTTG	22	54.4 - 58.8	56,6	4	36.3	12.0	N	-

Virus	Primer name	Sequence (5’→3’)	Lenght	Tm (°C)	Tm mean (°C)	Degeneracy	Hairpin	Self-dimer	Specificity (3 nt na 3’ conserved; max de 5 mismatches)	Amplicon (pb)
	DENV2R8	TCCYTCTCCYTCCATCTG	18	52.3 - 56.5	54,4	4	None	None	DENV1 (9/272), DENV3 (29/163), DENV4 (2/170), SLEV (2/31), WNV (22/126)	-
DENV3	DENV3F1	GGAGAATCYTCACCAAGYCCAAC	23	59.2 - 63.3	61,25	4	41.2	2.0	DENV1 (272/272), DENV4 (1/170)	-
	DENV3F2	AGAATCYTCACCAAGYCCAACAGTG	25	61.2 - 65.0	63,1	4	None	None	DENV2 (3/217), JEV (108/203)	-
	DENV3F3	CGACATGTYAATGCRGAACCA	21	56.9 - 61.5	59,2	4	37.3	24.9	N	653
	DENV3F4	CATGTYAATGCRGAACCAGAAACAC	25	59.4 - 63.3	61,3	4	30.8	0.4	-	649
	DENV3F5	GTYAATGCRGAACCAGAAACACCCA	25	62.0 - 65.9	63,9	4	None	3.4	-	647
	DENV3F6	CRGAACCAGAAACACCCAAYATGGA	25	61.0 - 65.4	63,2	4	41.3	None	-	640
	DENV3F6.1	GAACCAGAAACACCCAAYATGGA	23	58.7 - 61.3	60	2	41.3	None	DENV1 (2/272)	638
	DENV3F7	AYAAAACGTGGGCTTACCAYGGATC	25	61.4 - 65.7	63,5	4	75.3	33.5	N	541
	DENV3F8	TGGGCTTACCAYGGATCYTATGAAG	25	60.5 - 64.0	62,2	4	46.8	15.4	N	533
	DENV3F9	GCYACAGGCTCAGCCTCCTCCATGA	25	67.7 - 70.1	68,9	2	61.5	11.5	N	503
	DENV3F10	TGGAGRACCYTGGGAAGGAA	20	58.2 - 62.1	60,1	4	52.7	22.2	N	296
	DENV3F11	GGRCARAGAACGTGAACTC	19	54.0 - 58.2	56,1	4	None	8.9	DENV1 (4/272), DENV2 (2/217), DENV4 (10/170), MVEV (1/12), TBEV (1/137)	135
	DENV3F12	CAGTGGAGTRGAAGGRGAA	19	52.7 - 58.5	55,6	4	None	None	DENV1 (270/272), DENV2 (217/217), DENV4 (168/170), JEV (1/203), LANV (3/3), TBEV (32/137)	-
	DENV3F13	GGACAGGTRGGRACTTATGG	20	54.5 - 59.5	57	4	41.8	None	DENV1 (267/272), DENV2 (83/217), DENV4 (165/170)	-

Virus	Primer name	Sequence (5'→3')	Lenght	Tm (°C)	Tm mean (°C)	Degeneracy	Hairpin	Self-dimer	Specificity (3 nt na 3' conserved; max de 5 mismatches)	Amplicon (pb)
	DENV3R1	CTCTTATRGTTCT-GCTYTCTTCCAC	25	57.5 - 60.9	59,2	4	None	None	-	-
	DENV3R2	GTGTTTCTG-GTTCYGCATTRA-CATG	25	59.4 - 63.3	61,3	4	None	None	-	179
	DENV3R3	TGATCCTTYT-TATTCTTTC	19	45.3 - 46.6	45,9	2	None	None	JEV (1/203), SLEV (11/31), TBEV (23/137), YFV (1/40)	217
	DENV3R4	TCATCATAGTGC-CATGTTGA	20	54.4	54.4	1	None	1.6	N	251
	DENV3R5	TTCACRACTC-CRTTTATCATG	21	51.8 - 56.1	53,9	4	48.8	None	N	341
	DENV3R6	CTCTC-CACTCCTTTRK-TTTCC	21	53.6 - 58.2	55,9	4	37.9	None	-	-
	DENV3R7	GATGGCTGC-CATTGRGGTATG	21	59.1 - 61.4	60,2	2	45.3	32.9	N	-
DENV4										
	DENV4F1	TATGGYTGGAAY-TTGGTCAA	20	52.6 - 56.7	54,6	4	None	None	DENV3 (47/163), TBEV (96/(137)	-
	DENV4F2	GTCRGGAAA-CATYGTGAG	18	50.6 - 55.5	53,0	4	35.6	2.2	-	768
	DENV4F3	CACCARGA-AGGRAAATGT-GAATC	23	56.3 - 59.6	57,9	4	43.9	None	-	116
	DENV4F4	CARATGGAAGCW-GAAGGAG	19	53.6 - 55.0	56,6	4	35.4	None	-	-
	DENV4R1	CAYGATTCACATT-TYCCTTC	20	50.4 - 54.7	52,5	4	37.1	None	DENV2 (192/217)	713
	DENV4R2	AACCTRTC-GACRCCACACTC	20	57.0 - 62.7	59,8	4	None	19.6	N	-
ZIKV										
	ZIKVF1	TGGTGCAAAGC-TATGGGTGGAACA	24	64.9	64,9	1	None	None	MVEV (12/12), WNV (104/126), YFV (8/40), SPOV (1/1)	-
	ZIKVF2	GAGTCATCRTCY-AGTCCTGAAGTG	24	58.2 - 62.7	60,4	4	None	None	JEV (1/203), SPOV (1/1)	-
	ZIKVF3	CATAAARAGTGT-GTCCACCA	20	53.7 - 55.0	54,3	2	42.9	None	-	756

Virus	Primer name	Sequence (5'→3')	Lenght	Tm (°C)	Tm mean (°C)	Degeneracy	Hairpin	Self-dimer	Specificity (3 nt na 3' conserved; max de 5 mismatches)	Amplicon (pb)
	ZIKVF4	AAGCTGYGCT-GARGCTCC	18	57.6 - 62.8	60,2	4	50.3	18.3	N	642
	ZIKVF5	GTGACTGGAGTY-ACAGGAA	19	53.7 - 56.3	55	2	60.1	27.6	N	437
	ZIKVF6	AAAARGTGGA-CACYAGGGTGC-CAGA	25	64.2 - 68.2	66,2	4	53.6	23.4	N	370
	ZIKVF7	AGGGTGC-CAGAYCCYCAAGA	20	60.3 - 65.3	62,8	4	71.8	6.7	WNV (13/126)	356
	ZIKVF7.1	AGGGTGC-CAGAYCCYCAAG	19	58.7 - 63.9	61,3	4	71.8	6.7	N	356
	ZIKVF8	GCAATATTT-GAAGAG-GAAAAAGA	23	53.6	53.6	1	None	None	-	209
	ZIKVF9	TTGAAGAG-GAAAAAGARTG-GAAGAC	25	57.7 - 59.2	58,45	2	None	None	YFV (35/40)	202
	ZIKVF10	AGAGARCAY-CACCTGAGAG-GAGAG	24	60.9 - 64.8	62,85	4	41.3	None	MVEV (1/12), SLEV (26/31), SPOV (1/1)	125
	ZIKVF11	AAGGTYCTYA-GACCAGCTGAA	21	56.6 - 60.8	58,7	4	61.3	17.4	N	-
	ZIKVF12	TCCATTGTGGTYC-CYTGCCGCCAC	24	68.0 - 71.2	69,6	4	39.2	3.2	N	-
	ZIKVF13	CCRGGGGCRG-GATGGAGCA	19	64.0 - 69.2	66,6	4	36.1	None	N	-
	ZIKVR1	ATGTTC-CACCCATAGCTTT-GCACCA	25	65.0	65	1	None	None	TBEV (1/137), WNV (23/126)	-
	ZIKVR2	AGGAGCTGRC-TYGTGGTGGACA-CAC	25	66.6 - 70.3	68,4	4	58.1	5.7	N	80
	ZIKVR3	TCYTCATAY-TTCACTGGCCTCC	22	57.8 - 61.2	59,5	4	None	3.3	-	125
	ZIKVR4	TCAGCRCAGCT-TRCCACAGC	20	60.6 - 66.6	63,6	4	83.0	11.5	N	173
	ZIKVR5	GTCATRGC-TATTCCTG-TRACTCCA	24	57.6 - 62.2	59,9	4	45.2	12.1	N	395

Virus	Primer name	Sequence (5'→3')	Lenght	Tm (°C)	Tm mean (°C)	Degeneracy	Hairpin	Self-dimer	Specificity (3 nt na 3' conserved; max de 5 mismatches)	Amplicon (pb)
	ZIKVR6	CCTTCTTGRGGRT-CTGGCACC	21	60.4 - 65.0	62,7	4	49.8	None	N	470
	ZIKVR7	GTCTTCCAY-TCTTTTCCTCTTC	23	55.5 - 57.1	56,3	2	None	None	MVEV (12/12), YFV (37/40)	626
	ZIKVR8	GGTGGCGGCARG-GRACCACAATGGA	25	69.7 - 72.7	71,2	4	63.0	12.0	N	-
	ZIKVR9	CARG-CAGTCTCCC-GRATGC	19	58.9 - 62.7	60,8	4	55.3	7.3	N	-

N: not tested.

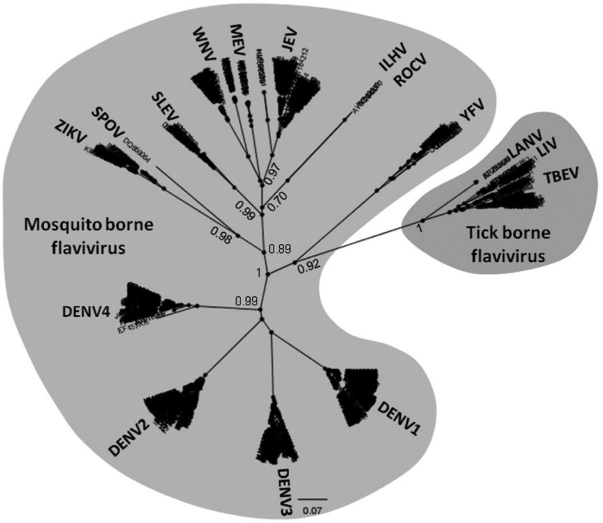

Phylogenetic reconstruction of Flavivirus subgroups using 800-806 bp sequences of the NS5 variable region. Phylogenetic relationships were inferred using MEGA7 software (Kumar et al. 2016), adopting the Neighbor-Joining method (Saitou & Nei 1987) and the Tajima-Nei model (Tajima & Nei 1984). The rate variation among the sites was predicted with gamma distribution (shape parameter = 1). The analysis was performed from an alignment of 1,442 nucleotide sequences of 800 nt in length, corresponding to the variable region of the NS5 protein. All positions containing gaps and missing data were disregarded. The numbers correspond to the bootstrap values and branch lengths for the number of base substitutions per site. All calculations were conducted using the MEGA7 software. The rate variation among sites was predicted with a gamma distribution (shape parameter = 1).
